# Construction of an organelle-like nanodevice via supramolecular self-assembly for robust biocatalysts

**DOI:** 10.1186/s12934-018-0873-3

**Published:** 2018-02-20

**Authors:** Hongxia Li, Guojun Zheng, Shaozhou Zhu

**Affiliations:** State Key Laboratory of Chemical Resources Engineering, Beijing University of Chemical Technology, 100029 Beijing, People’s Republic of China

**Keywords:** Nanodevice, Self-assembly, (+)-γ-lactamase, Biocatalysis, Nanocompartments, Synthetic biology, Protein dodecahedron, Nanoreactor

## Abstract

**Background:**

When using the microbial cell factories for green manufacturing, several important issues need to be addressed such as how to maintain the stability of biocatalysts used in the bioprocess and how to improve the synthetic efficiency of the biological system. One strategy widely used during natural evolution is the creation of organelles which can be used for regional control. This kind of compartmentalization strategy has inspired the design of artificial organelle-like nanodevice for synthetic biology and “green chemistry”.

**Results:**

Mimicking the natural concept of functional compartments, here we show that the engineered thermostable ketohydroxyglutarate aldolase from *Thermotoga maritima* could be developed as a general platform for nanoreactor design via supramolecular self-assembly. An industrial biocatalyst-(+)-γ-lactamase was selected as a model catalyst and successful encapsulated in the nanoreactor with high copies. These nanomaterials could easily be synthesized by *Escherichia coli* by heterologous expression and subsequently self-assembles into the target organelle-like nanoreactors both in vivo and in vitro. By probing their structural characteristics via transmission electronic microscopy and their catalytic activity under diverse conditions, we proved that these nanoreactors could confer a significant benefit to the cargo proteins. The encapsulated protein exhibits significantly improved stability under conditions such as in the presence of organic solvent or proteases, and shows better substrate tolerance than free enzyme.

**Conclusions:**

Our biodesign strategy provides new methods to develop new catalytically active protein-nanoreactors and could easily be applied into other biocatalysts. These artificial organelles could have widely application in sustainable catalysis, synthetic biology and could significantly improve the performance of microbial cell factories.

**Graphical Abstract:**

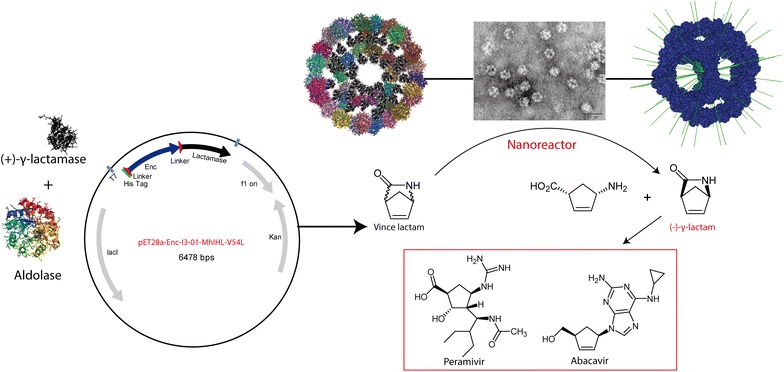

**Electronic supplementary material:**

The online version of this article (10.1186/s12934-018-0873-3) contains supplementary material, which is available to authorized users.

## Background

Cells have evolved diverse compartments to organize metabolic pathways [1]. The well-defined barriers formed by lipid membranes or proteins constrain certain incompatible chemical processes in the nano-spatial scale [1–3]. This brings several advantages, such as: (a) increasing the local concentrations of enzymes and substrates; (b) protecting the unstable catalysts or volatile intermediates from unsuitable environments; and (c) creating unique microenvironments for catalysis [4, 5]. These features make them excellent models for chemical process optimization and provide inspiration for biomimetic design.

Natural protein based nano-compartments are becoming increasingly attractive as materials for wide applications such as nanoreactors, therapeutic delivery systems, and advanced functional materials synthesis [6, 7]. For example, the carboxysome are bacterial origin, organelle-like protein cage with a diameter of about 100 nm [8]. In each carboxysome, two kinds of enzymes (ribulose-1,5-bisphosphate carboxylase–oxygenase and carbonic anhydrase) are encapsulated in the protein cage to enhance CO_2_ fixation [8, 9]. Furthermore, encapsulins, protein cages with a diameter of about 30 nm, are involved in important processes like iron mineralization [5, 10]. These intriguing creations make nature a master of the art and have initiated new efforts in bioinspired chemistry. Compared with most of other inorganic/organic nanostructures, proteinaceous nanostructures exhibit many superior properties and represent a unique combination of chemistry, biology and materials science [11]. Generally, they have robust self-assembly properties and are soluble in aqueous solvents. Moreover, they are biological origin which makes them inherently biocompatible and allows for precise control [11]. For instance, intensive studies have been performed on virus capsids, and these versatile nanoparticles have been widely used in nanobiotechnology nowadays. Douglas et al. designed a biomolecular catalyst for hydrogen production based on the self-assembling properties of bacteriophage P22 capsids [12]. Through a scaffold protein, the [NiFe]-hydrogenase was successfully embedded within the viral protein coat, which provides a strong protection to the protein cargo and increase the catalytic efficiency [12]. Another example is ferritin, which has been used to encapsulate rhodium nanoclusters for organic synthesis [13]. Recently discovered Encapsulin from *Thermotoga maritima* was demonstrated that can be used as a targeted delivery system for therapeutic medicines [14]. Besides a growing number of these natural origin protein-based compartments, several artificial virus-like protein nanoparticles have been designed [15, 16]. For example, a hyperstable 60-subunit protein dodecahedron was created by Baker et al., which was directly self-assembled from an engineered trimeric protein called ketohydroxyglutarate aldolase from *T. maritima* [15]. Later on, another two components, 120-subunit icosahedral protein nanocage was also created by the same group [16]. These impressive designs have attracted considerable attention, and repurposing these protein compartments for applications ranging from nanoreactor design to drug delivery is of great interest and is currently underway.

To manipulate these fascinating protein-microcompartments, three general strategies have been developed: (1) fusion of the cargo protein to a scaffold that directs encapsulation to the nanocage interior; (2) specific packaging directly by natural or artificial sequence, e.g. SpyTag/SpyCatcher; and (3) nonspecific packaging by self-assembly [11]. Generally, the last two strategies have obvious disadvantages: yields of the encapsulated protein products are low. This is due to the efficiency of the self-assembly and the limited interior space of the protein cage. On the other hand, genetic fusion is more challenging yet could be precisely designed. In this study, we report the use of the protein nanocage as a platform for nanoreactor design. We hypothesized that encapsulation of the enzyme in the interior of a protein nanocompartment might improve both enzyme activity and stability which was proved by a (+)-γ-lactamase as the model biocatalyst. Moreover, it is envisioned that the protein nanoreactor, which can encapsulate diverse biocatalysts via genetic fusions, might provide a robust and universal method for the encapsulation of highly active biocatalysts, which might have wide application in biocatalysis and “green manufacturing”.

## Results

### Preparation of recombinant proteins for nanoreactor construction

**Fig. 1:**
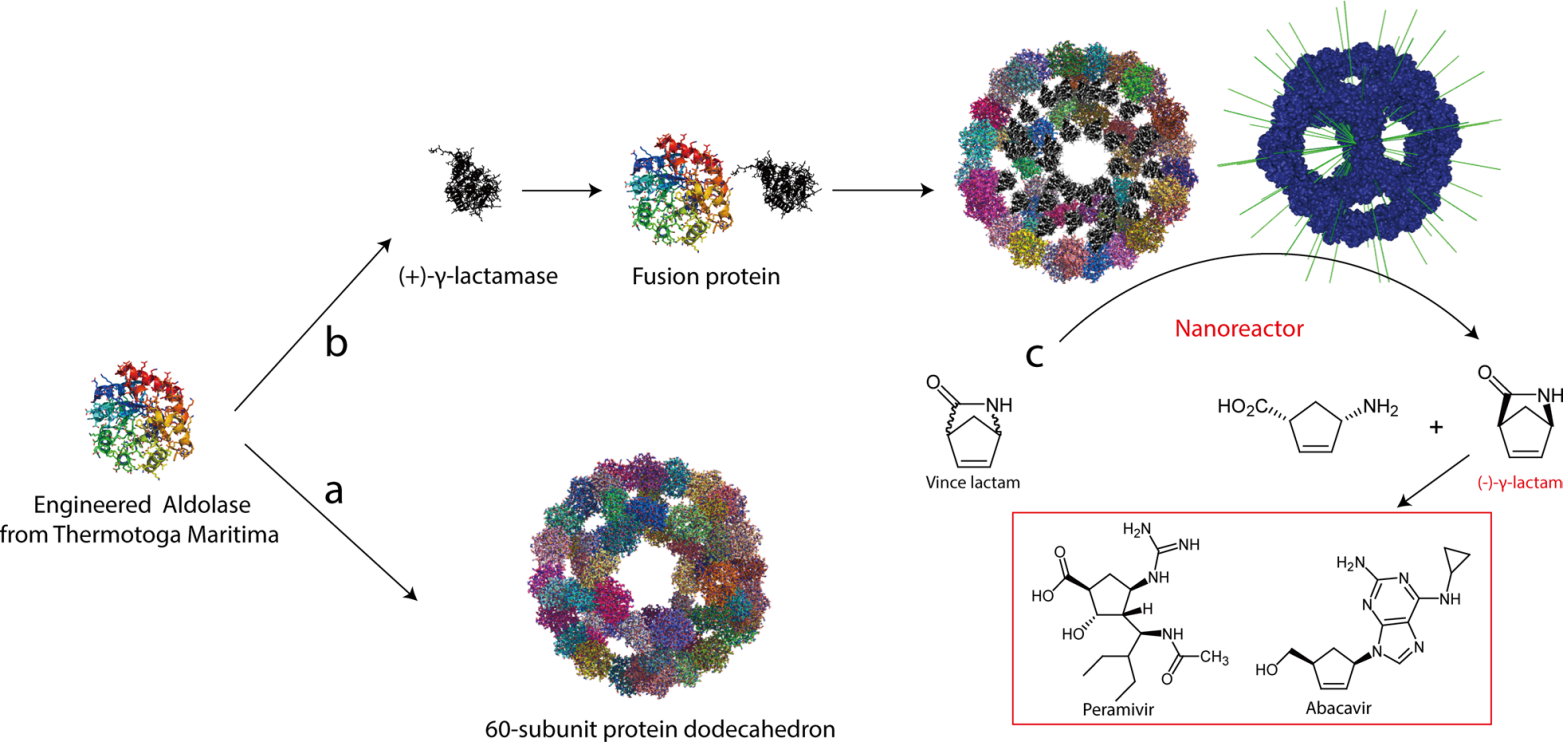
Strategy for self-assembly of γ-lactamase Nanoreactor. (*a*) The engineered aldolase from *T. maritima* could self-assembles into a dodecahedron protein nanocage. (*b*) γ-lactamase was fused at the C terminus of the coating protein and self-assembles into a nanoreactor. (*c*) Scheme showing the reaction catalyzed by the nanoreactor

**Fig. 2:**
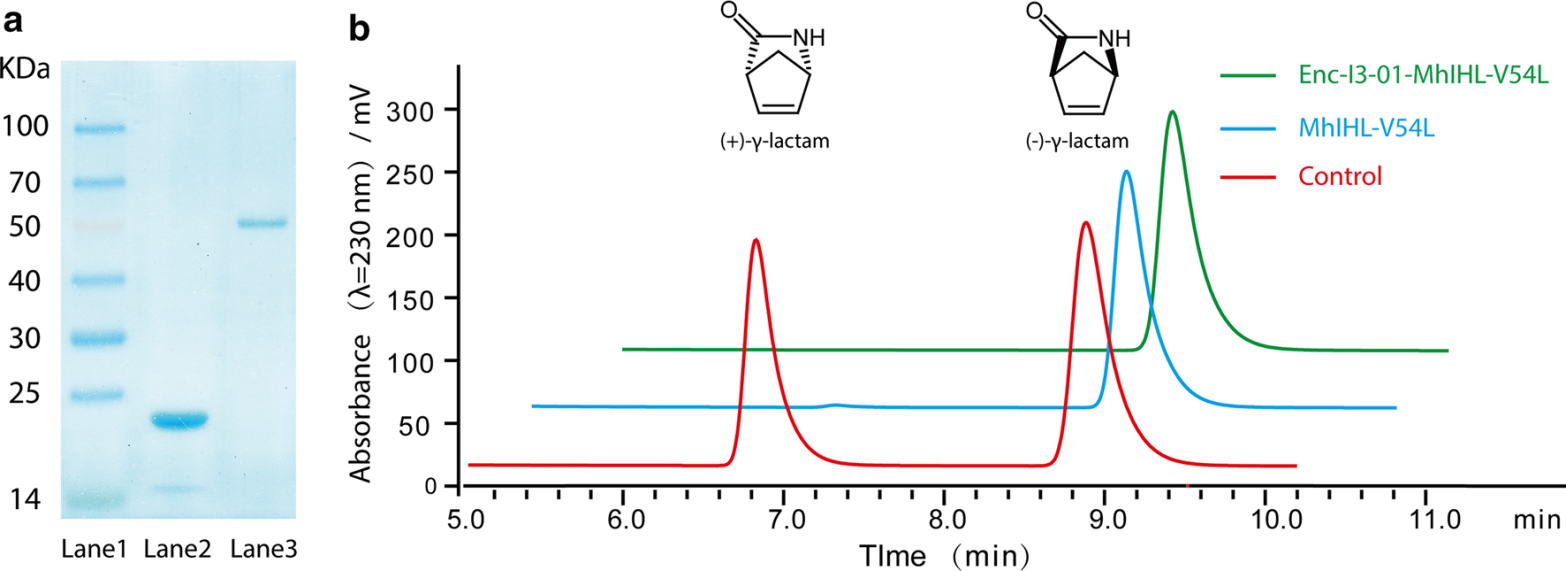
Preparation of free and encapsulated γ-lactamases and their bioactivities. **a** SDS-PAGE of purified γ-lactamase. Lane 1, protein marker; lane 2, purified free γ-lactamase; lane 3, purified encapsulated γ-lactamase. **b** Chiral-HPLC analysis of biocatalytic reaction by γ-lactamase

The engineered ketohydroxyglutarate aldolase from *T. maritima* can self-assemble into a virus-like dodecahedron particle with 60 subunits (Fig. 1) [15]. Based on RosettaDesign, the trimeric aldolase with newly optimized interfaces between each building blocks has been showed that it could self-assemble into dodecahedron protein assemblies both in vivo and in vitro without any treatments [15]. The nanoparticles are remarkably stable at high concentration of guanidine hydrochloride and high temperature (up to 80 °C) [15]. To expand the application of such a protein nanocage, we have herein used the dodecahedron to develop novel nanoreactors. A γ-lactamase mutant (designated as MhIHL-V54L hereafter) from *Microbacterium hydrocarbonoxydans* was selected as a model biocatalyst and placed in the interior of the nanocage. γ-Lactamase is a versatile enzyme that is widely used for the enzymatic resolution of racemic 2-azabicyclo [2.2.1] hept-5-en-3-one (Vince lactam) [17–21]. The chiral products are valuable synthons used for an increasing number of drug candidates, such as Ziagen (Abacavir) [19]. Past research has shown that the lactamase from *M. hydrocarbonoxydans* had a superior activity among all reported (+)-γ-lactamases [22]. However, one shortcoming of this biocatalyst is its thermolabile nature, which hinders further industrial application [22]. To construct a nanoreactor for this unstable lactamase (Fig. 1), the lactamase was first cloned into pET28a, and a single mutation (Val54Leu) was introduced to improve its enantioselectivity, resulting in a pET-28a-MhIHL-V54L plasmid [22]. Next, the engineered ketohydroxyglutarate aldolase from *T. maritima* (designated as Enc-I3-01 hereafter) was cloned with a His_6_ tag encoded at the N terminus to facilitate purification and a short linker encoded at the C terminus to separate the two domains (Additional file 1: Fig. S1). The synthesized Enc-I3-01 was then cloned into pET-28a-MhIHL-V54L vector by *Nco*I and *Nde*I, resulting in the pET28a-Enc-I3-01-MhIHL-V54L plasmid. After sequencing, the right plasmids were transformed into *E. coli* BL21(DE3) and induced by IPTG for heterologous expression. Recombinant proteins were then purified by Ni–NTA affinity chromatography. These enzymes were concentrated and further purified by a size-exclusion chromatography step. The purity of the obtained proteins was analyzed by SDS-PAGE. As shown in Fig. 2a, SDS-PAGE analysis revealed a single band with the correct apparent molecular weight. Size-exclusion chromatography showed that the fusion protein had an unusually short retention time, indicating that it might form a supramolecular structure (Additional file 1: Fig. S2).

### Bioactivity and structural characterization of nanocaged enzymes

To determine the bioactivity of the constructed nanoreactor, the encapsulated (+)-γ-lactamase and the free enzyme were then assayed with the Vince lactam as the substrate in the typical assay described in the methods section. The activity of each enzyme was determined by chiral HPLC. The results show that the mutated free (+)-γ-lactamase exhibited high enantioselectivity (99%) and specifically hydrolyzed the (+)-γ-lactam. On the other hand, its fusion with the engineered ketohydroxyglutarate aldolase did not affect its bioactivity. The encapsulated (+)-γ-lactamase also showed high enantioselectivity and activity, indicating that encapsulation did not affect its function (Fig. 2b).

**Fig. 3:**
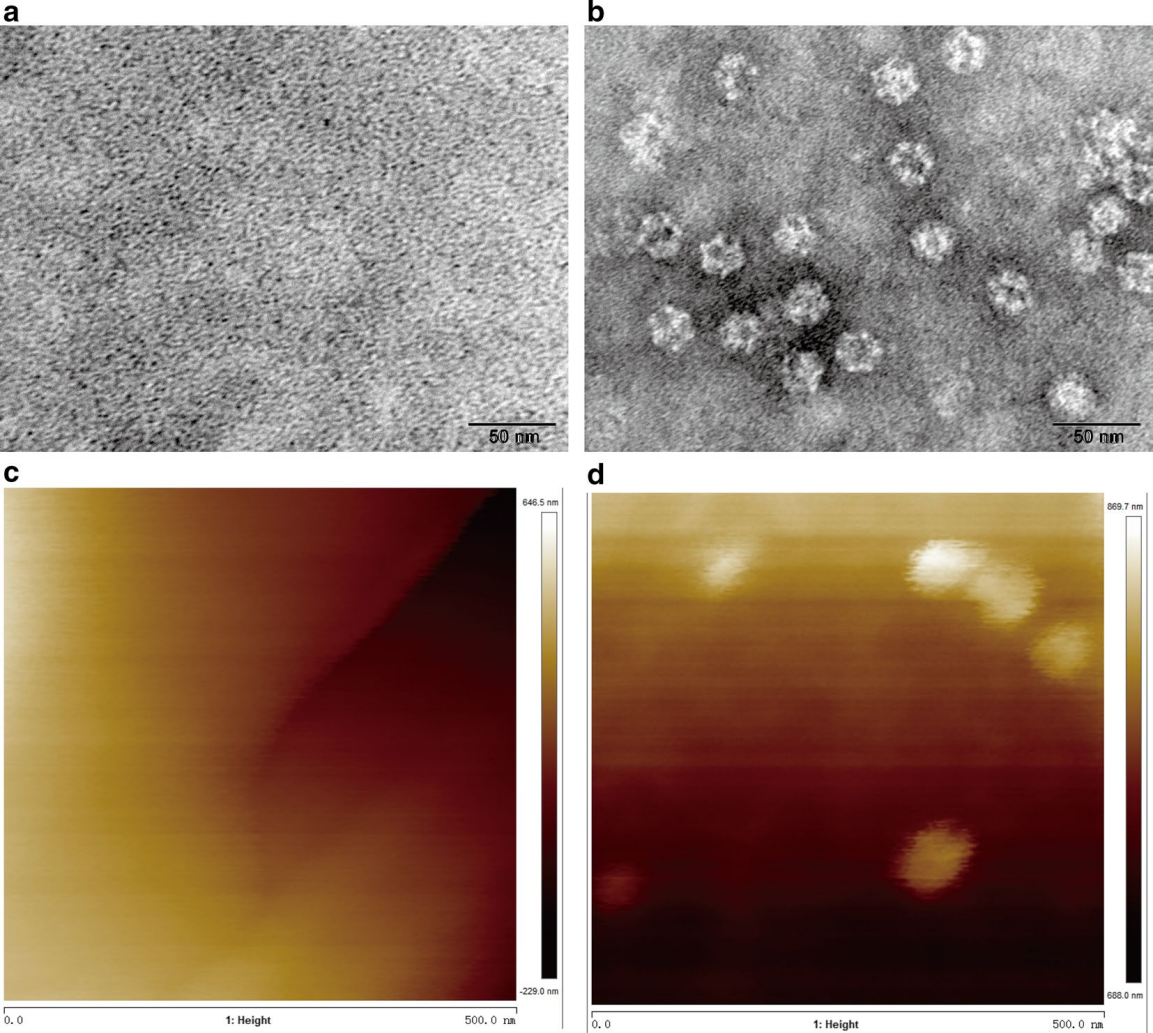
Structures of free γ-lactamase and encapsulated γ-lactamase self-assembled in vitro. Representative images from TEM: **a** free γ-lactamase; **b** encapsulated γ-lactamase. Representative images from AFM: **c** free γ-lactamase; **d** encapsulated γ-lactamase

**Fig. 4:**
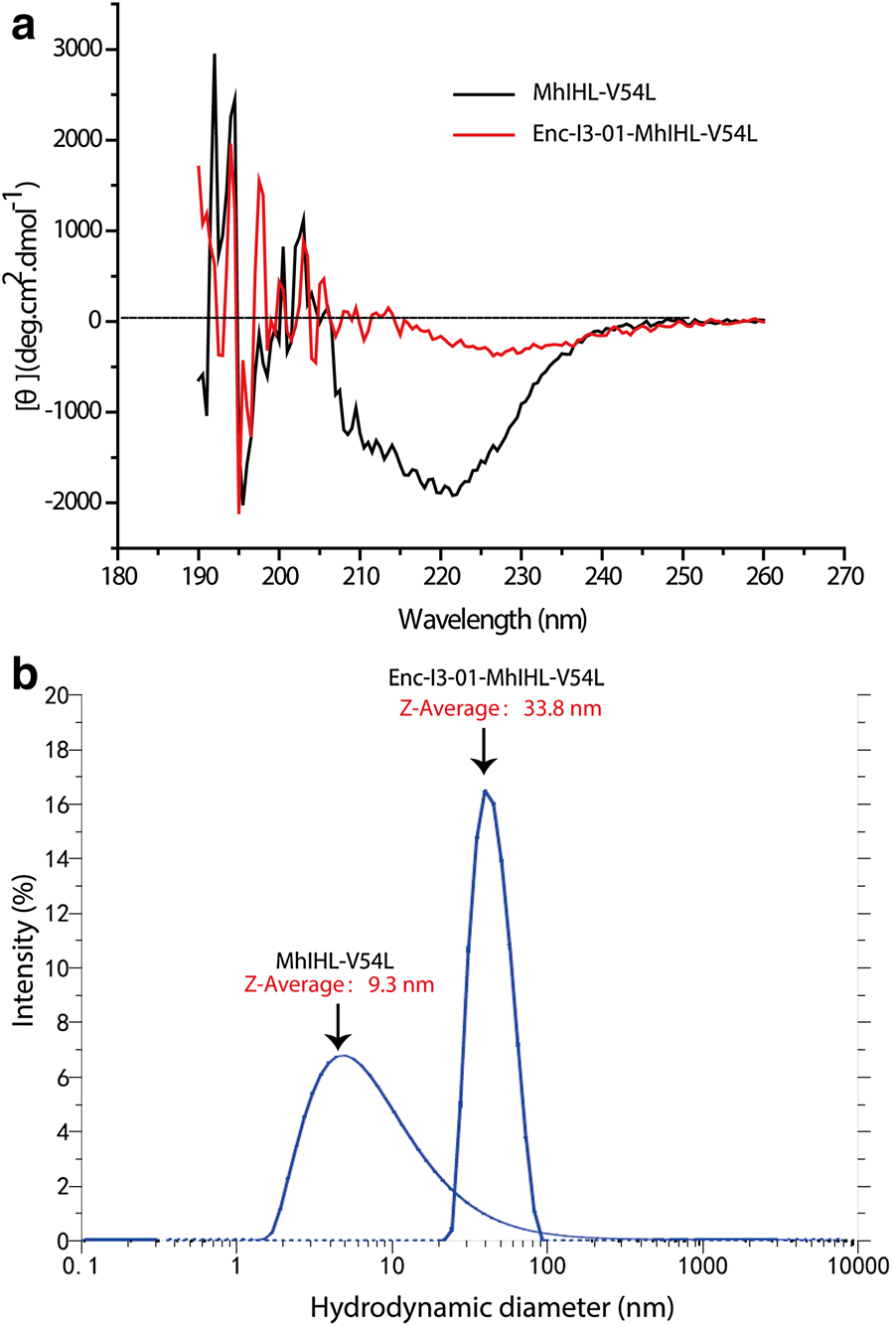
CD spectra and DLS measurements of free γ-lactamase and encapsulated γ-lactamase. **a** CD spectra of free and encapsulated γ-lactamase. **b** DLS measurement of hydrodynamic diameter of free and encapsulated γ-lactamase

To prove that the fusion proteins could indeed be assembled into a dodecahedron nanoreactor, high-resolution transmission electron microscopy (TEM) was applied to probe the structure of the protein cage. TEM showed that the free (+)-γ-lactamase did not form any obvious 3D structure (Fig. 3a, Additional file 1: Fig. S3a). Conversely, the fusion protein had a typical virus-like structure (Fig. 3b, Additional file 1: Fig. S3b). The individual particles were homogenous in terms of size and shape, clearly indicating that they form the designed structure: a dodecahedron with a diameter of about 30 nm and an interior volume of approximately 3000 nm^3^ [15]. Considered that only 60 subunits could form such kind of 30 nm dodecahedron structures, it could be deduced that 60 copies of the cargo lactamases theoretically encapsulated in a single cage [15]. Atomic force microscopy (AFM) analysis supported the same conclusion. While the free (+)-γ-lactamase had no obvious structure (Fig. 3c, Additional file 1: Fig. S4a), the fusion protein formed nanoreactors of about 30 nm in height (Fig. 3d, Additional file 1: Fig. S4b), in agreement with the predicted size. We further used circular dichroism spectra to probe the globular protein secondary structure and used dynamic light scattering to probe the size of the nano-particles (Fig. 4a, b). As expected, the free and encapsulated enzymes produce very different spectra in CD analysis indicating these two proteins have different protein secondary structure. Notably, the fusion protein does not show strong secondary structure for a typical protein. This might due to its special structure where high copies of enzymes were concentrated in the interior of the nanocage. The arrangement of the encapsulated cargo proteins in the nanocage might not be very orderly. On the other hand, the dynamic light scattering diameter of the encapsulated lactamase is in the range of 30–35 nm which further proved the fusion protein could form the designed structure. Taken together, these results confirm the (+)-γ-lactamase was successfully embedded in the interior of the nanocages, with 60 copies of the enzyme theoretically encapsulated in a single cage based on computer modeling (Additional file 1: Fig. S5).

### Compare the properties of the free and encapsulated (+)-γ-lactamase

From a chemical perspective, encapsulation strategy could enhance the performance of biocatalyst. First, the coat protein can protect the cargo proteins from harsh environment. More important, it is possible that 60 copies of enzymes concentrated in a confined space might significantly improve the catalytic efficiency. Past research has shown that encapsulation enzymes in inorganic/organic material or immobilization on solid supports could confer several benefits to biocatalysts, such as enhancing stability as well as facilitating re-use [23]. Compared with other strategies where the enzymes are sequestered in or on a wide variety of nonbiological compartments, sequestered enzymes in biological compartments like ours might be a superior strategy since no further processing is required. To show that the protein nanocages could confer benefits to the enzymes, biochemical characterization of the free and encapsulated (+)-γ-lactamase were performed using Vince lactam as the substrate. First, Km and Kcat for each enzyme were measured and interpreted from the Lineweaver–Burk plots. The results showed that Michaelis–Menten parameters for both biocatalysts are very similar. They are 120.4 ± 7.2 mM and 20,088 ± 718 s^−1^ for the free MhIHL-V54L and 86 ± 2.6 mM and 12,830 ± 164.5 s^−1^ for the encapsulated MhIHL-V54L, respectively (Additional file 1: Fig. S6). These data indicate that protein coat insignificantly affects the performance of these biocatalysts, especially the transport of the substrate to MhIHL-V54L and the release of the product.

**Fig. 5:**
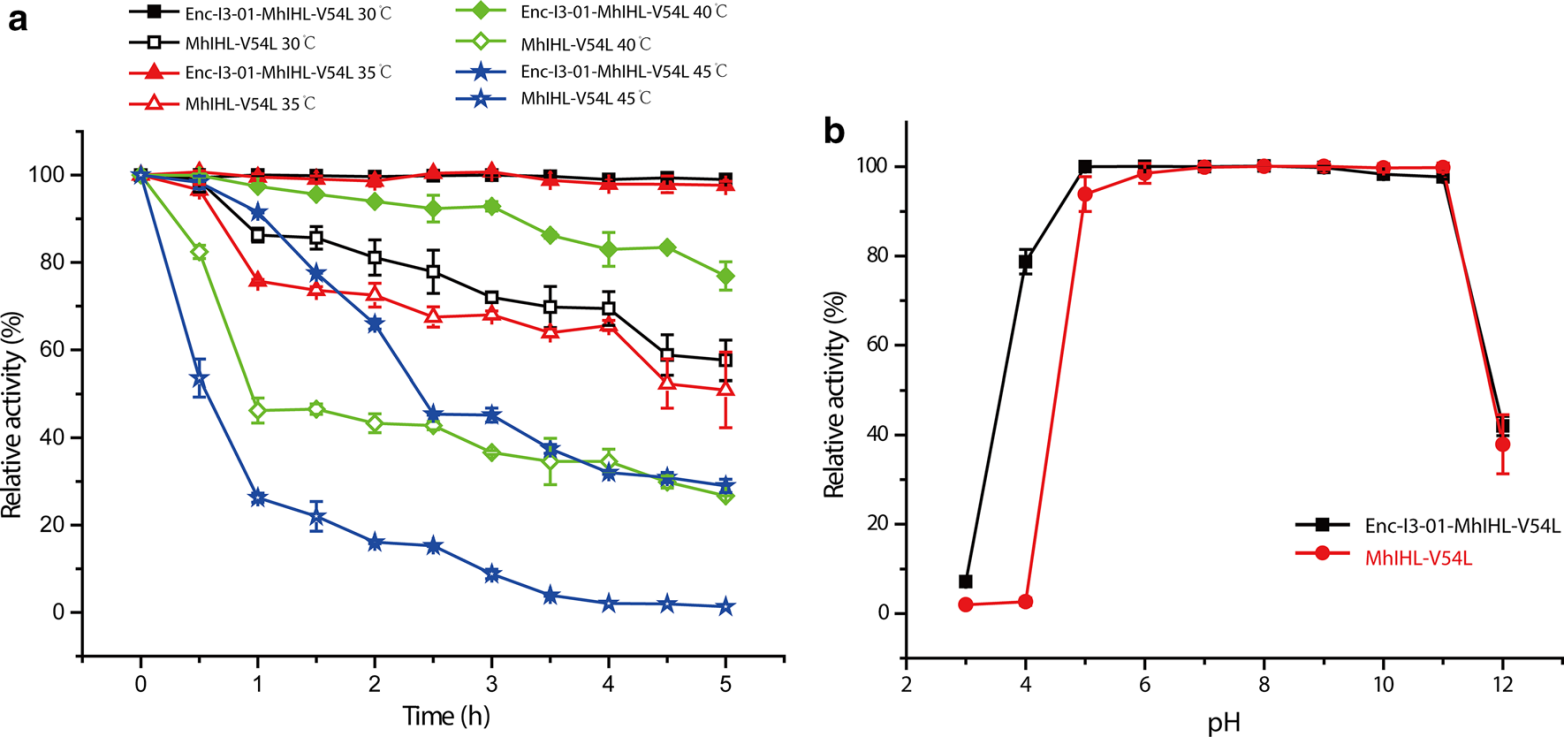
Biochemical characterization of free and encapsulated γ-lactamase. **a** Comparison of the thermostability of the free and encapsulated γ-lactamase. **b** Comparison of the pH stability of the free and encapsulated γ-lactamase

We then probed the optimal temperature for both the free and encapsulated (+)-γ-lactamase. Interestingly, we found that the optimal temperature for the free enzyme was 15 °C while the optimal temperature for the encapsulated enzyme was 30 °C (Additional file 1: Fig. S7). This shift indicated that the encapsulated (+)-γ-lactamase might have a higher thermostability. To examine the thermostability of these two enzymes, both catalysts were treated at different temperature for various times in the assay buffer without any substrate, and reactions were then initiated by adding the substrate at 20 °C for 20 min. Our data showed that the free lactamase already lost half of its activity at 35 °C after 5 h, and almost lost all its initial activity at 45 °C after 5 h, indicating that it is a very thermolabile enzyme (Fig. 5a). In contrast, the encapsulated enzyme maintained almost 100% of its initial activity at 35 °C for 5 h. It only started to lose its activity above 40 °C, and only lost 20% of its activity after 5 h. These results suggest that forming such dodecahedral nanocompartment could significantly improve the stability of the cargo enzyme. This improvement might due to the compact structure of the nanocage, which hinders thermal fluctuations of each enzymes. Moreover, all the enzymes are immobilized on the interior surface of the nanocage which further increase the stability of cargo proteins.

The impact of pH on the enzyme activity was also investigated. The encapsulated (+)-γ-lactamase has similar optimal pH to the free enzyme (Additional file 1: Fig. S8). However, pH stability studies showed that the encapsulated enzymes performed better at pH 4. It retained 80% of its initial activity compared with the unprotected enzyme, which completely lost its activity under such conditions (Fig. 5b). These results also suggest that the microcompartments could create a unique microenvironment regarding pH for the enzymes and confer pH stability to the enzymes.

### Nanocaged enzymes are protected from polar organic solvents deactivation and proteolysis

**Fig. 6:**
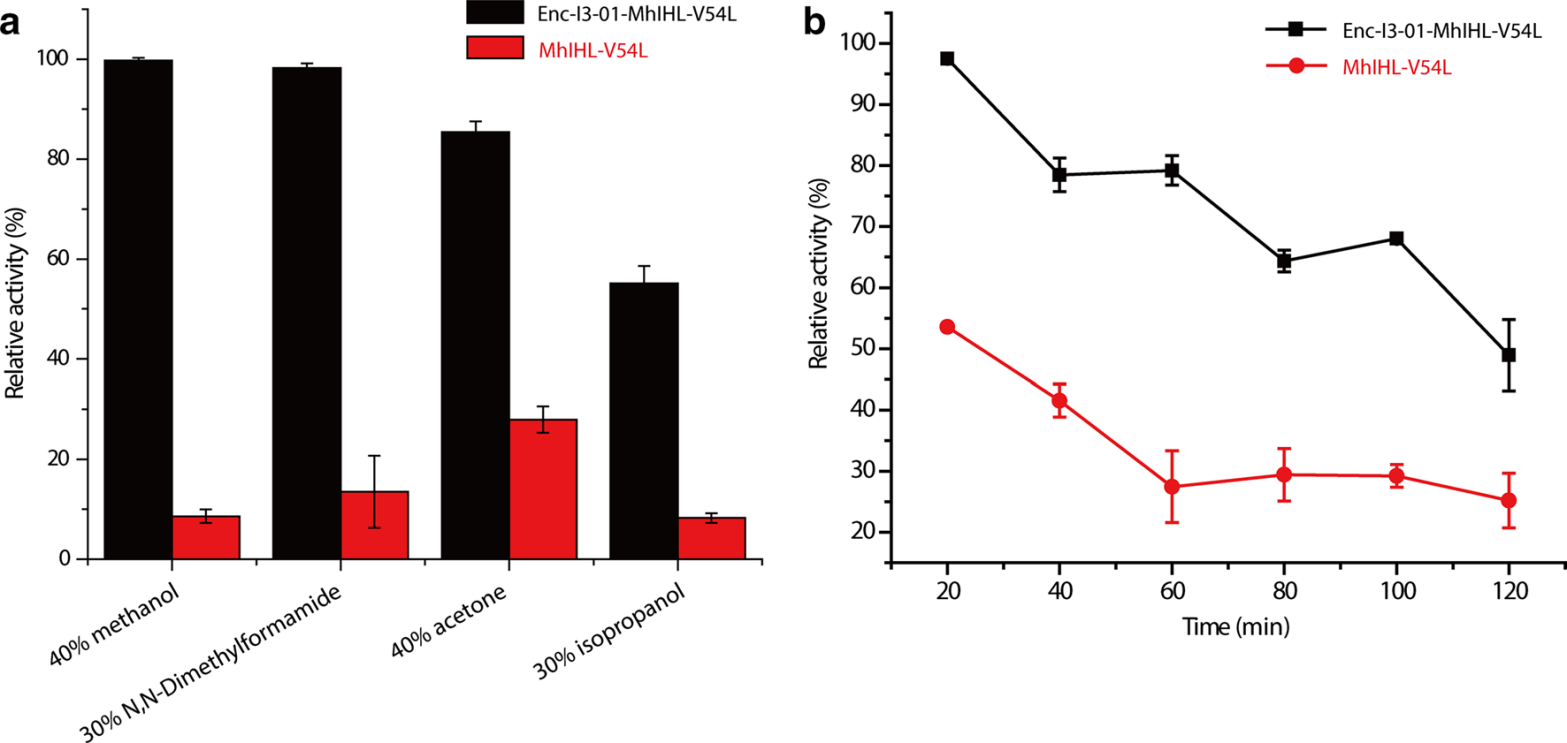
Biochemical characterization of free and encapsulated γ-lactamase. **a** Comparison of the stability of the free and encapsulated γ-lactamase in presence of organic solvents. **b** Comparison of the stability of the free and encapsulated γ-lactamase in presence of protease (1:50)

Past research showed that enzymes encapsulated in silica, polymer or nanogel had better stability in polar organic solvents [23]. Enzymes encapsulated in protein nanocage might have a similar affect. To test the effect of the polar organic solvent, several organic solvents were selected to treat the model enzymes. 40% methanol, 30% *N*,*N*-dimethylformamide, 40% acetone, and 30% isopropanol were used to treat the free and the encapsulated lactamase at 4 °C for 10 min, respectively. The residual activity was then measured. As shown in Fig. 6a, the encapsulated (+)-γ-lactamase maintained over 99, 99, 80, and 50% of its initial activity, respectively. On the contrast, the free enzyme had a completely different performance and lost almost all initial activity. We guess that the nanocage might be able to create a small hydrophilic environment for the cargo protein which could contribute to the significantly increased stability in organic solvent. It is also possible that the nanocage could reserve some water that is necessary for the enzymes to carry out their biological functions in the interior of the cage. On the other hand, without this kind of protection, the essential water for free enzymes was easily stripped by solvents, which led to denaturation of the unprotected enzyme.

Other nanocages such as self-assembled DNA nanocages were previously found to protect the encapsulated enzymes against proteases [24]. We guess that the protein nanocage should have a similar function. As shown in Additional file 1: Fig. S9, a time-course experiment revealed that the encapsulated (+)-γ-lactamase was highly resistant to degradation performed by trypsin (1:10), and maintained more than 90% of its initial activity after treated with trypsin for 5 h (Additional file 1: Fig. S9). In contrast, the free (+)-γ-lactamase only retained 40% of its initial activity at the same condition. We then increased the amount of trypsin (1:50) used in the assays. As showed in Fig. 6b, the encapsulated (+)-γ-lactamase still performed much better than the free one. The encapsulated lactamase retained 50% of its initial activity after 2 h. This is probably because the protein coat could also be degraded by proteases. Nevertheless, these results demonstrate that the protein nanocage protects the encapsulated enzymes from protease degradation.

### Nanocaged enzymes have stronger tolerance against high substrate concentrations

**Fig. 7:**
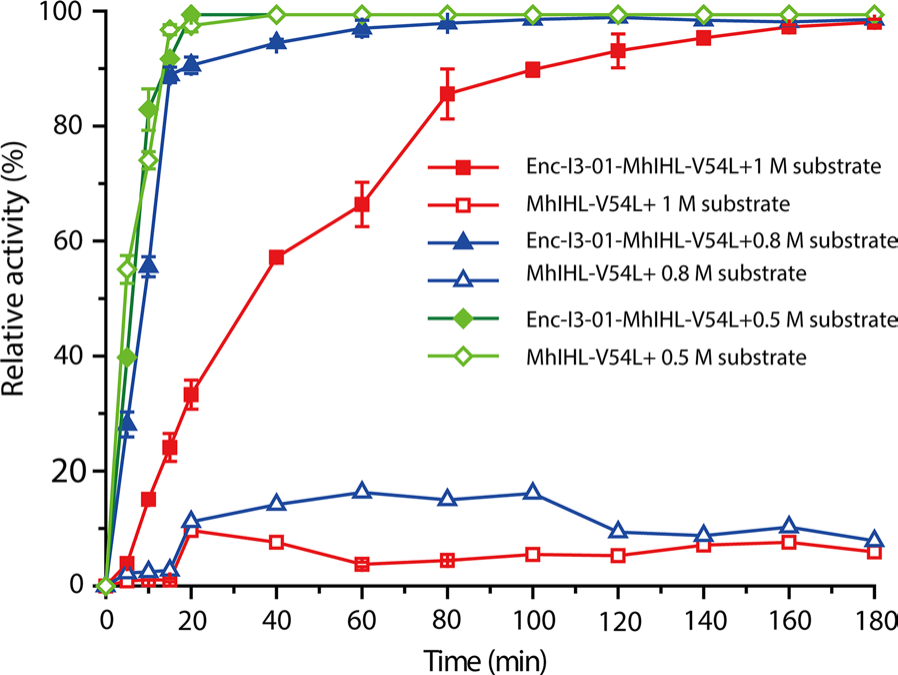
Comparison of the substrate tolerance of the free and encapsulated γ-lactamase

Enzymes’ tolerance ability against high substrate concentrations is an important issue in developing efficient biocatalysts in synthetic biology [25, 26]. The advantage brought by this feature is that it could significantly reduce the product separation cost and facilitate the re-use of biocatalysts. Therefore, the substrate tolerance of the free and encapsulated (+)-γ-lactamases was investigated at three different concentrations of substrate (Fig. 7). Interestingly, when we used a lower concentration of substrate, the free (+)-γ-lactamase (only 0.1 μM biocatalysts) had a similar or better performance compared to the encapsulated enzyme. However, when we increased the concentration of the substrate to 0.8 M, free enzyme could only weakly hydrolyze the Vince lactam. On the other hand, this had little effect on the activity of the encapsulated (+)-γ-lactamase (Fig. 7). When we further increased the concentration to 1.0 M, the encapsulated (+)-γ-lactamase could also be inhibited; however, it could still slowly hydrolyze the (+)-γ-lactam and reached a 99% ee value after 3 h (Fig. 7). To further investigate the inhibitory effect of substrate, experiments were carried out individually by varying the concentration of substrate. As shown in Additional file 1: Fig. S10, the Lineweaver–Burk plot clearly showed that the reaction rate is inhibited by higher substrate concentrations for both the free and encapsulated lactamase. However, nanocaged enzymes have stronger tolerance against high substrate concentrations than the free enzymes. We propose two explanations for this phenomenon. First, the protein nanocage provides a specific environment for catalysis wherein it controls substrate access and concentration used in the nanocage, thus avoiding inhibition at high concentrations of substrate. Second, it is possible that covalent attachment to the protein shell stabilizes the structure of the encapsulated (+)-γ-lactamase, making it active even after 3 h at high concentration of substrate. These results are also consistent with the thermostability studies, further demonstrating that construction of an organelle like our self-assembling nanoreactor provides efficient access to robust biocatalysts.

## Discussion

In natural evolution, one of the biggest differences between eukaryotes and prokaryotes is that eukaryotes have created diverse organelles for compartmentalization of metabolic pathway which is a hallmark of higher cells [27]. Prokaryotes, on the contrast, have evolved only some organelle like protein nanocompartments such as ferritin, encapsulin and the carboxysome [1, 3]. Generally, these compartmentalization strategies could bring great advantages to the cells such as keeping the high fidelity of metabolic pathway, improving the efficiency of enzymatic reaction and reducing the toxicity of metabolites to cells [7]. Currently, prokaryotes like *E. coli* or yeast is the most widely used “microbial cell factories” for synthetic biology due to their clear genetic background, high gene expression level and simple culture conditions. Thus, the ability to create and redesign protein containers for new synthetic purposes in these systems present an unprecedented opportunity for creating nanofactories. Self-assembled proteins with cage-like architectures, such as carboxysome and virus capsids, exist widely in nature [1]. Diverse artificial protein nanocages have also been designed [15, 16]. All these materials provide particularly powerful platforms for the development of novel nanoreactors and nanofactories.

Past researches have showed that couples of strategies have been described for the encapsulation of active enzymes. However, they could only be used for in vitro studies. For example, Ouyang et al. used nanogel to encapsulate enzymes which was shown that can improve the biocatalytic activity and stability of biocatalysts [23]. Zhao et al. developed a DNA assembling strategy for enzymes encapsulation and have a similar effect [24]. All these strategies can only be used after the enzymes were purified and cannot be applied for in vivo studies. On the contrast, compartments based on protein-building blocks could be developed into nanofactories both in vivo and in vitro in a precise fashion. Compared with other studies, the work presented here shows several advantages. (1) The cargo protein or biocatalysts could be directly fused with the coat protein, resulting in high copies of catalyst per nanoreactor (60 copies per nanocage). When using other protein nanocage as a nanoreactor, a specific tag must normally be employed to mediate encapsulation of the cargo protein, and copy numbers are low. Moreover, two or more separated proteins need to be prepared to accomplish the encapsulation which make the system complex and inaccurate. (2) Compared with other encapsulation by inorganic/organic nanomaterial, this strategy does not require downstream treatment that could destroy part of the cargo protein. (3) This strategy could significantly improve the stability of the cargo protein and make the biocatalyst superior to its native form. Moreover, it is envisioned that this system could also be used to encapsulate multiple enzymes and develop multistep enzyme cascades nanoreactor. All these make it a promising tool to develop new generation of nanofactories for synthetic biology.

## Conclusion

In conclusion, we herein established an efficient and simple method to develop an organelle-like nanoreactor based on protein self-assembling. The engineered ketohydroxyglutarate aldolase from *T. maritima* could be developed as a universal nano-platform for diverse biocatalysts. The cargo biocatalyst of interest could be easily fused to the C terminus of the engineered aldolase and encapsulated into the microcompartments with high copy number. Using one model biocatalyst—(+)-γ-lactamases, we showed that these unique nanoparticles could protection the enzyme system and improve the catalytic performance. Compared with the free enzyme, the encapsulated lactamase exhibits similar biocatalytic behaviour as evidenced by a similar Km and Kcat, but significantly improved thermostability at high temperature and in the presence of proteases, organic solvent and high substrate load. Thus, our studies provide a general compartmentalization strategy for biocatalysis, synthetic biology and metabolic engineering. We believe that such uniform nanoreactor at nanoscale could find a large variety of application in “Green Chemistry” and “Microbial Cell Factories”.

## Methods

### Chemicals and bacterial strains

Q5 DNA polymerase and T4 DNA ligases were purchased from NEB (New England Biolabs). Oligonucleotides of HPLC purity were synthesized by Sangon Biotech (China). The gene for the engineered ketohydroxyglutarate aldolase (Genebank Accession Number: WP_010865041.1) from *T. maritima* was synthesized by Synbio Technologies (China). The gene was cloned into the pUC57 vector and was subsequently used as a template for PCR reactions. All primers used in this study are listed in Additional file 1: Table S1. Gel Extraction Kits and Plasmid Mini Kits were purchased from OMEGA (USA). Other chemicals were purchased from commercial sources and were of analytical grade.

### Cloning and protein purification

The gene sequence encoding for (+)-γ-lactamase from *M. hydrocarbonoxydans* (Genebank Accession Number: AKS37009) was amplified by PCR from the genomic DNA of *M. hydrocarbonoxydans* and DNA polymerase. The primers used are shown in Additional file 1: Table S1. Purified PCR products and pET-28a(+) plasmid were digested with *Nde*I and *Xho*I restriction endonucleases and ligated with T4 DNA ligase to yield an final expression plasmid (named as pET-28a-MhIHL). Mutagenesis of MhHL was achieved using specific primers listed in Additional file 1: Table S1. The yield plasmid was named as pET-28a-MhIHL-V54L. The engineered ketohydroxyglutarate aldolase from *T. maritima* was amplified by PCR. The purified products and plasmid pET-28a-MhIHL-V54L were then digested with *Nde*I and *Nco*I and ligated with T4 DNA ligase to construct the final expression plasmid (pET28a-Enc-I3-01-MhIHL-V54L) for the nanoreactor.

The DNA ligation products were then transformed into *E. coli* DH5*α* cells and individual transformants were cultured to prepare plasmids. After confirmed by DNA sequencing, the resulting expression constructs were used to transform *E. coli* BL21(DE3) cells and cultured in LB (lysogeny broth) medium at 37 °C with shaking overnight. 50 µg/mL of kanamycin were added to the medium to maintain the plasmid. From these cultures, 0.5-L baffled culture flasks, each containing 200 mL of LB media, were inoculated to OD_600_ ≈ 0.01. The culture was then grown at 37 °C to an OD_600_ of 0.7 and was placed in a refrigerator for 30 min to cool down. The cells were induced after adding 0.05 mM of IPTG (isopropyl thiogalactopyranoside) and shaken for overnight. All the cells were then harvested via centrifugation (6000 rpm for 10 min). The cell pellets were then suspended in Tris–HCl buffer and lysed by sonication. Supernatant of the lysate was separated by centrifugation and loaded on a Ni–NTA gravity flow column. After a washing step, recombinant proteins were eluted from the Ni–NTA column and combined for further size exclusion chromatography (Superdex 200 column, GE Healthcare). After loaded on the column, protein-containing fractions were collected, concentrated and flash-frozen using liquid N_2_. The protein concentrations were determined by using a Thermo NanoDrop 2000.

### Enzyme assays

0.5 M (rac)-γ-lactam was dissolved in a universal buffer (50 mM Tris, 50 mM boric acid, 33 mM citric acid and 50 mM Na_2_PO_4_, pH was adjusted by NaOH or HCl). for in vitro studies. In a typical assay, ≈ 0.1 μM biocatalysts was incubated with 0.5 M substrates in 500 μL assay buffer. The reaction was performed at 20 °C for 20 min and quenched by addition of TFA (0.1% (v/v)). 20 μL of the reaction solution was then extracted with ethyl acetate (1000 μL). The bioactivity of the free and encapsulated (+)-γ-lactamase was determined by chiral HPLC.

### Analytical methods

10 μL of the ethyl acetate extract was then subjected to HPLC. The separation of racemic lactam was achieved by a Daicel Chiralpak AS-H column (Japan) with the following gradient:90% acetonitrile and 10% isopropanol (volume ratio, 1.1 mL/min). The UV absorbance used for detection was 230 nm. The ee and conversion (conv.) were calculated as follows:

$$ee\,\left(\% \right) = \frac{{\left| {{\text{S}_{\text{a}}} - {\text{S}_{\text{b}}}} \right|}}{{{\text{S}_{\text{a}}} + {\text{S}_{\text{b}}}}} \times 100\%$$

$$\text{Conversion}\,\left( \% \right) = \frac{{\left( {{\text{C}}_{ 0} - {\text{C}}} \right)}}{C} \times 100\%$$

where S_a_ and S_b_ are represented for the final concentration of each γ-lactam; C_0_ and C were the initial and final concentrations of γ-lactam in the reaction.

### Negative-stain electron microscopy

TEM (Transmission electron microscopy) data was acquired on a Hitachi H-800 transmission electron microscope. One drop of purified MhIHL-V54L and Enc-I3-01-MhIHL-V54L at 1.33 mg/mL were adsorbed onto a copper grids. After a short adsorption time, the protein samples were stained with 3% uranyl acetate for 0.5 h. Samples were ready for TEM analysis after all the excess liquid was removed. Grids were optimized for data collection and final data collection was performed on a 120 kV JEM1200-EX transmission electron microscope.

### Atomic force microscope

For atomic force microscopy, samples were prepared by dropping 5 μL of purified MhIHL-V54L and Enc-I3-01-MhIHL-V54L at 1.33 mg/mL onto freshly leaved mica for 10 min and drying under air. Images were acquired using a scanning probe microscope (Bioscope Catalyst^®^ AFM, Bruker) operated in tapping mode. Height data analysis was performed using the Nanoscope 1.5 software package.

### Circular dichroism spectra and dynamic light scattering (DLS) measurements

CD analysis was conducted on a JASCO J-810 CD. The integration time was set to 4 s and the bandwidth was set to 1 nm. About 2.5 mL 1.33 mg/mL purified MhIHL-V54L and Enc-I3-01-MhIHL-V54L were prepared for CD assay. Ellipticity data of for both the free and encapsulated (+)-γ-lactamase was then continuously collected in the far-UV CD band of 190–260 nm. The spectrum of a buffer was selected as a blank and subtracted. For DLS measurements, purified protein (about 1.33 mg/mL) was measured using a SZ-100 Nanoparticle Analyzer (HORIBA). Both the free and encapsulated lactamases were measured at 25 °C. Measurements were taken in the universal buffer (pH 7.0).

### Comparison of enzymatic properties

Biochemical characterization was performed using the standard assays as mentioned above. The kinetic parameters of free and encapsulated (+)-γ-lactamase were determined by using different concentration of Vince lactam (20–250 mM) while keeping buffer and enzyme concentrations constant, and done in triplicate. The reactions were performed in 500 μL of assay buffer (pH 7.0) at optimum temperature for 3 min and were quenched by addition of TFA. Lineweaver–Burk plot was used to determine the kinetic parameters. Standard reaction systems were used to determine the optimal temperature for the free and encapsulated (+)-γ-lactamases. ≈ 0.1 μM biocatalysts was incubated with 0.5 M substrates in 500 μL assay buffer (pH 7.0) at different temperature for 5 min (from 10 to 50 °C). The thermostability of the free and encapsulated enzymes were investigated by incubation of enzymes in 250 μL assay buffer (pH 7.0) without substrate at 30–45 °C for 0.5, 1.0, 1.5, 2.0, 2.5, 3.0, 3.5, 4.0, 4.5, 5.0 h, respectively. The treated enzymes (final concentration: 0.1 μM) were then assayed with another 250 μL assay buffer (pH 7.0) containing 1 M substrates (final concentration: 0.5 M) at 20 °C for 20 min. The reaction was then quenched by addition of TFA (0.1% (v/v)). The residual activity of the treated enzymes was then measured by HPLC. The control was the one without any heat treatment.

To determine the optimal pH for the free and encapsulated (+)-γ-lactamases, reactions with different initial pH values ranging from 3.0 to 12.0 were formulated with 0.1 μM enzymes and 0.5 M substrates. The reaction was performed at 20 °C for 5 min and quenched by addition of TFA. Resulting mixtures were then measured by HPLC as described before. The pH stability of the free and encapsulated enzymes was investigated by incubation of enzymes in 50 μL assay buffer with different pH values (3–12) for 30 min at 4 °C. The treated enzymes (final concentration: 0.1 μM) were then assayed with another 450 μL assay buffer (pH 7.0) containing 1 M substrates (final concentration:0.5 M) at 20 °C for 20 min. The reaction was then quenched by addition of TFA and then measured by HPLC. Control was the one without any pH treatment.

To test the effect of polar organic solvent, the free and the encapsulated (+)-γ-lactamases were treated with 40% methanol, 30% *N*,*N*-dimethylformamide, 40% acetone, 30% isopropanol, separately. The assays were performed at 4 °C for 10 min and the treated enzymes (0.1 μM) were then assayed with another 450 μL assay buffer (pH 7.0) containing 1 M substrates (final concentration:0.5 M) at 20 °C for 20 min. The reaction was then quenched by addition of TFA and analyzed by HPLC. To assess the proteolytic stability of the free and encapsulated (+)-γ-lactamases, assays were performed using 1 μg of purified enzymes and two different concentration of Trypsin. The assays were performed with 0.2 μg/μl of trypsin (1:10) or 1 μg/μl of trypsin (1:50) in a 50 mM Tris–HCl buffer at pH 7.0 for different time (1–6 h) at 4 °C. The treated enzymes (final concentration: 0.1 μM) were then assayed with another 450 μL assay buffer containing 1 M substrates (final concentration: 0.5 M) at 20 °C for 20 min. The reaction was then quenched by addition of TFA and analyzed by HPLC as described before.

To assess the substrate tolerance ability of the free and encapsulated (+)-γ-lactamase, assays were performed at 3 different substrate concentrations. Generally, in a typical assay, 0.1 μM biocatalysts were incubated with 0.5, 0.8 or 1 M substrates in 500 μL assay buffer (pH 7.0). The reaction was incubated at 20 °C for different time (0, 5, 10, 15, 20, 40, 60, 80, 100, 120, 140, 160, 180 min, respectively) and quenched by TFA as described before. All the activity was analyzed by HPLC. All experiments were conducted with three sets of parallel. To investigate the inhibitory effect the substrate, experiments were performed by varying the concentration of the substrate. 0.1 μM biocatalysts were incubated with substrates in 500 μL assay buffer (pH 7.0). The reaction was incubated at 20 °C for 3 min and quenched by TFA. The Lineweaver–Burk double reciprocal plot was used to study the inhibitory effect of substrates.

## Additional file

**Additional file 1: Table S1.** Primers for cloning, mutagenesis of the MhIHL and for construction of the nanoreactor. **Fig. S1.** Schematic and sequence of artificially fused protein open reading frame for γ-lactamase nanoreactor. **Fig. S2.** Size-exclusion chromatography (SEC) of the free and encapsulated (+)-γ-lactamases. **Fig. S3.** TEM structures of free γ-lactamase and encapsulated γ-lactamase self-assembled in vitro. **Fig. S4.** AFM structures of free γ-lactamase and encapsulated γ-lactamase self-assembled in vitro. **Fig. S5.** Structures of empty protein dodecahedron formed by the engineered ketohydroxyglutarate aldolase from Thermotoga maritima. **Fig. S6.** Michaelis–Menten plot used to determine the Km and kcat values. **Fig. S7.** Optimal temperature for free γ-lactamase and encapsulated γ-lactamase. **Fig. S8.** Optimal pH for free γ-lactamase and encapsulated γ-lactamase. **Fig. S9**. Comparison of the stability of the free and encapsulated γ-lactamase in presence of protease. **Fig. S10.** The Lineweaver–Burk double reciprocal plot for different concentrations of (+)-γ-lactam.

## Abbreviations

AFM: atomic force microscopy
CD: circular dichroism spectra
DLS: dynamic light scattering
HPLC: high-performance liquid chromatography
MhIHL: lactamase from *Microbacterium hydrocarbonoxydans*
NTA: nitrilotriacetic acid
PCR: polymerase chain reaction
SDS-PAGE: sodium dodecyl sulfate polyacrylamide gel electrophoresis
SEC: size-exclusion chromatography
TEM: transmission electron microscopy
