# Supplementary material for: Construction of an organelle-like nanodevice via supramolecular self-assembly for robust biocatalysts
Source: Microb Cell Fact. 2018 Feb 20;17:26. doi: 10.1186/s12934-018-0873-3 (PMC5819227; doi:10.1186/s12934-018-0873-3)
Supplement: Supplementary file 1 — Additional file 1: Table S1. Primers for cloning, mutagenesis of the MhIHL and for construction of the nanoreactor. Fig. S1. Schematic and sequence of artificially fused protein open reading frame for γ-lactamase nanoreactor. Fig. S2. Size-exclusion chromatography (SEC) of the free and encapsulated (+)-γ-lactamases. Fig. S3. TEM structures of free γ-lactamase and encapsulated γ-lactamase self-assembled in vitro. Fig. S4. AFM structures of free γ-lactamase and encapsulated γ-lactamase self-assembled in vitro. Fig. S5. Structures of empty protein dodecahedron formed by the engineered ketohydroxyglutarate aldolase from Thermotoga maritima. Fig. S6. Michaelis–Menten plot used to determine the Km and kcat values. Fig. S7. Optimal temperature for free γ-lactamase and encapsulated γ-lactamase. Fig. S8. Optimal pH for free γ-lactamase and encapsulated γ-lactamase. Fig. S9. Comparison of the stability of the free and encapsulated γ-lactamase in presence of protease. Fig. S10. The Lineweaver–Burk double reciprocal plot for different concentrations of (+)-γ-lactam. [file 12934_2018_873_MOESM1_ESM.docx]

**Construction of an organelle-like nanodevice via supramolecular self-assembly for robust biocatalysts**

**Additional Information**

**Content**

| 1 | **Table. S1** Primers for cloning, mutagenesis of the MhIHL and for construction of the nanoreactor | S2 |
| --- | --- | --- |
| 2 | **Fig. S1** Schematic and sequence of artificially fused protein open reading frame for γ-lactamase nanoreator | S3 |
| 3 | **Fig. S2** Size-exclusion chromatography (SEC) of the free and encapsulated (+)-γ-lactamases | S4 |
| 4 | **Fig. S3** TEM structures of free γ-lactamase and encapsulated γ-lactamase self-assembled in vitro | S5 |
| 5 | **Fig. S4** AFM structures of free γ-lactamase and encapsulated γ-lactamase self-assembled in vitro | S6 |
| 6 | **Fig. S5** Structures of empty protein dodecahedron formed by the engineered ketohydroxyglutarate aldolase from Thermotoga maritima. | S6 |
| 7 | **Fig. S6** Michaelis-Menten plot used to determine the Km and kcat values | S7 |
| 8 | **Fig. S7** Optimal temperature for free γ-lactamase and encapsulated γ-lactamase | S7 |
| 9 | **Fig. S8** Optimal pH for free γ-lactamase and encapsulated γ-lactamase | S8 |
| 10 | **Fig. S9** Comparison of the stability of the free and encapsulated γ-lactamase in presence of protease | S8 |
| 11 | **Fig. S10** The Lineweaver–Burk double reciprocal plot for different concentrations of (+)-γ-lactam | S9 |

**Table S1.** Primers for cloning, mutagenesis of the MhIHL and for construction of the nanoreactor. The restriction enzyme cutting sites are underlined. Mutated positions are highlighted in bold.

| constructs | name | sequence |
| --- | --- | --- |
| pET-28a-MhIHL | MhIHL_FP | GGAATTCCATATGGCAGCACCCCGCCGCACCGTCGTCCT |
|  | MhIHL_FP | CCGCTCGAGTCAGAGAGCGACGTGGTCGTGCGTGGCGAT |
| pET-28a-MhIHL-V54L | MhIHL-V54L_FP | CATCACGATCCCTTGGGAGTCGGCA |
|  | MhIHL-V54L_RP | GGCGTGCCGACTCCCAAGGGATCGTG |
| pET28a-Enc-I3-01-MhIHL-V54L | Enc-I3-01-FP | CATGCCATGGGCCATCATCATCATCATCACGGCGGCAGC |
|  | Enc-I3-01-RP | GGAATTCCATATGGCTGCCGCTGCC GCTGCCGCTG |

## Figure


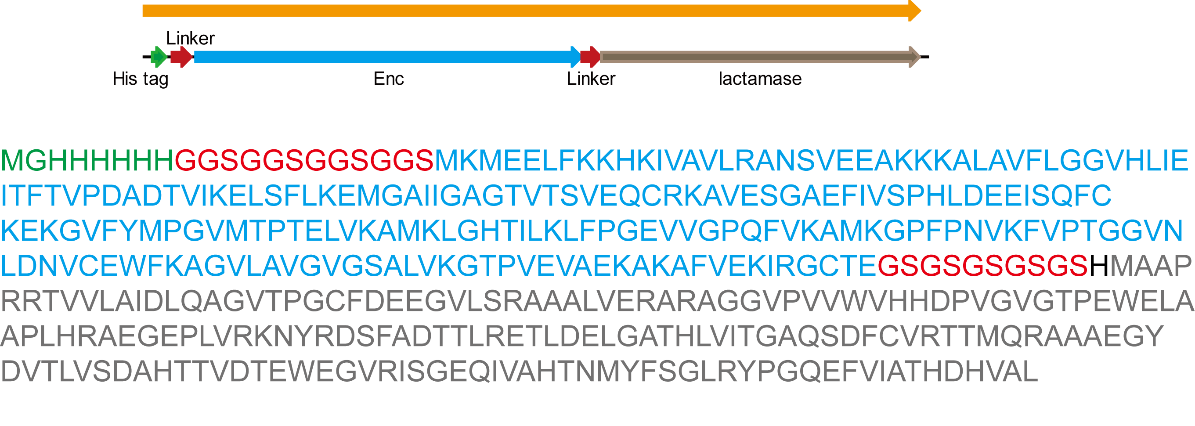


**Fig. S1** Schematic of artificially fused protein open reading frame for γ-lactamase nanoreator (top); Sequences of the artificially fused protein. The fusion protein contains a His tag (green), two linkers (red), coat protein (blue) and the (+)-γ-lactamase(brown).


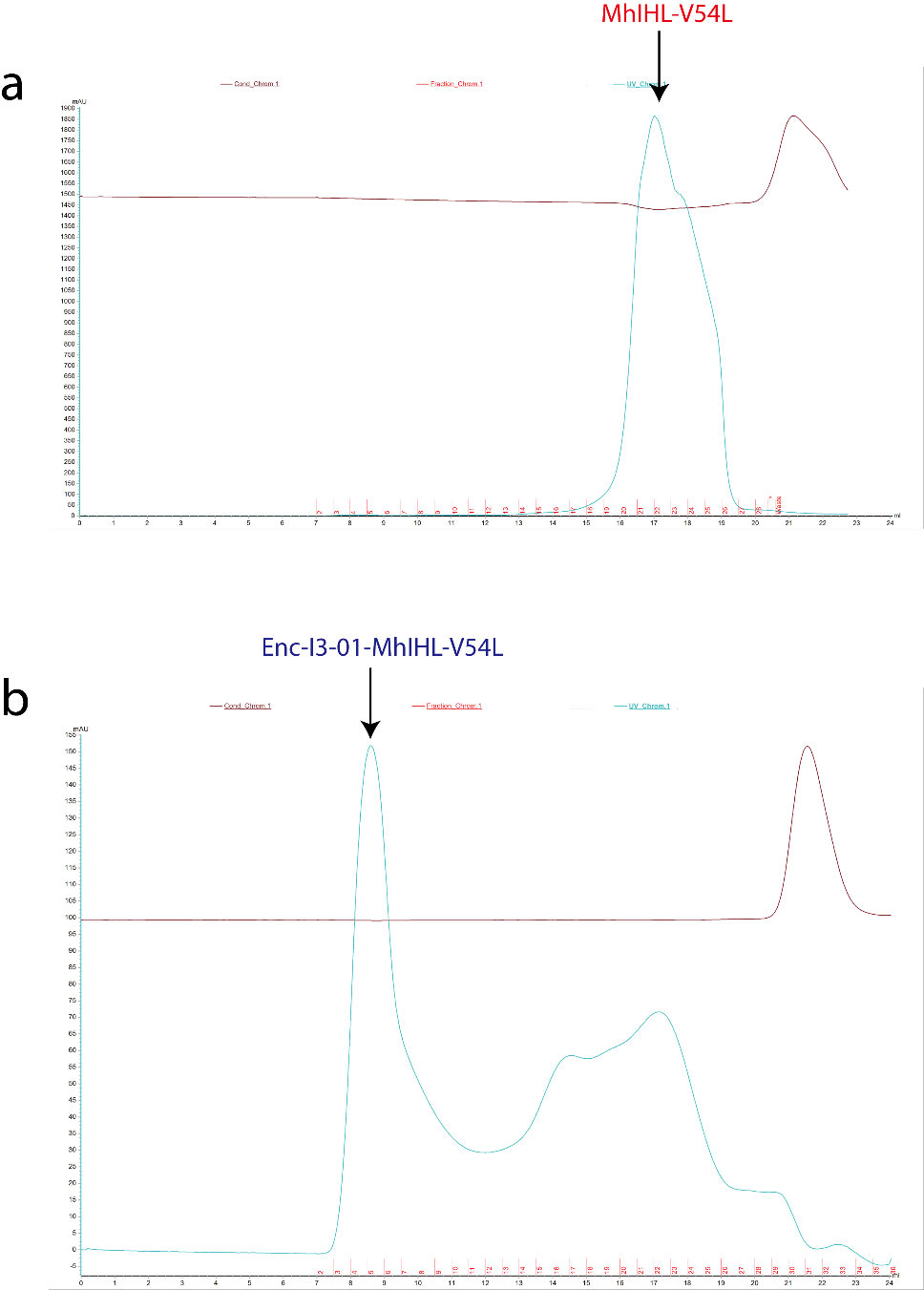


**Fig. S2** Size-exclusion chromatography (SEC) of the free and encapsulated (+)-γ-lactamases. SEC was monitored by absorbance at 280 nm. (a) the free (+)-γ-lactamase, MhIHL-V54L; (b) encapsulated γ-lactamase, Enc-I3-01-MhIHL-V54L.


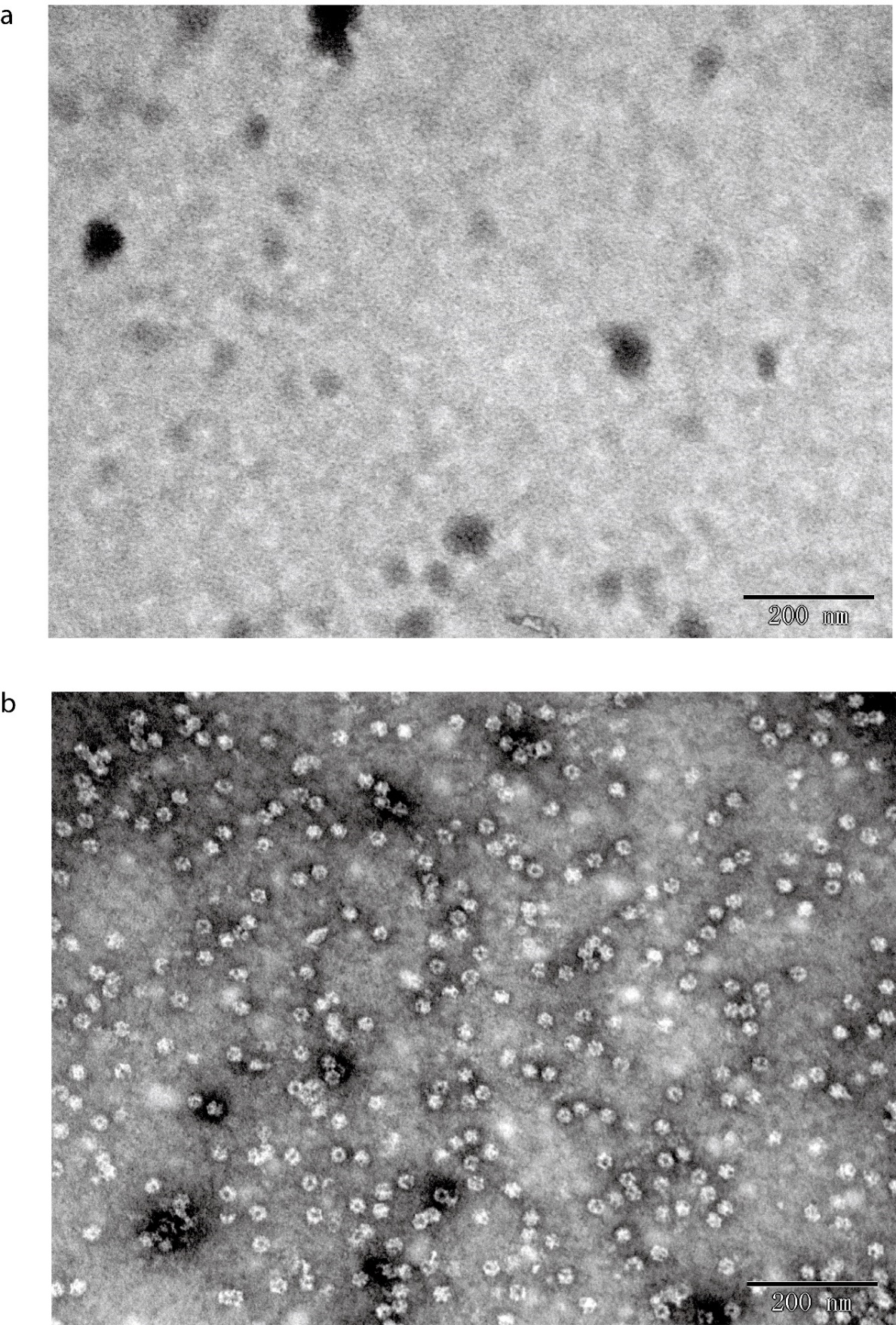


**Fig. S3** Structures of free γ-lactamase and encapsulated γ-lactamase self-assembled in vitro. Representative

images from TEM (200 nm): a) free γ-lactamase, MhIHL-V54L; b) encapsulated γ-lactamase, Enc-I3-01-MhIHL-V54L.


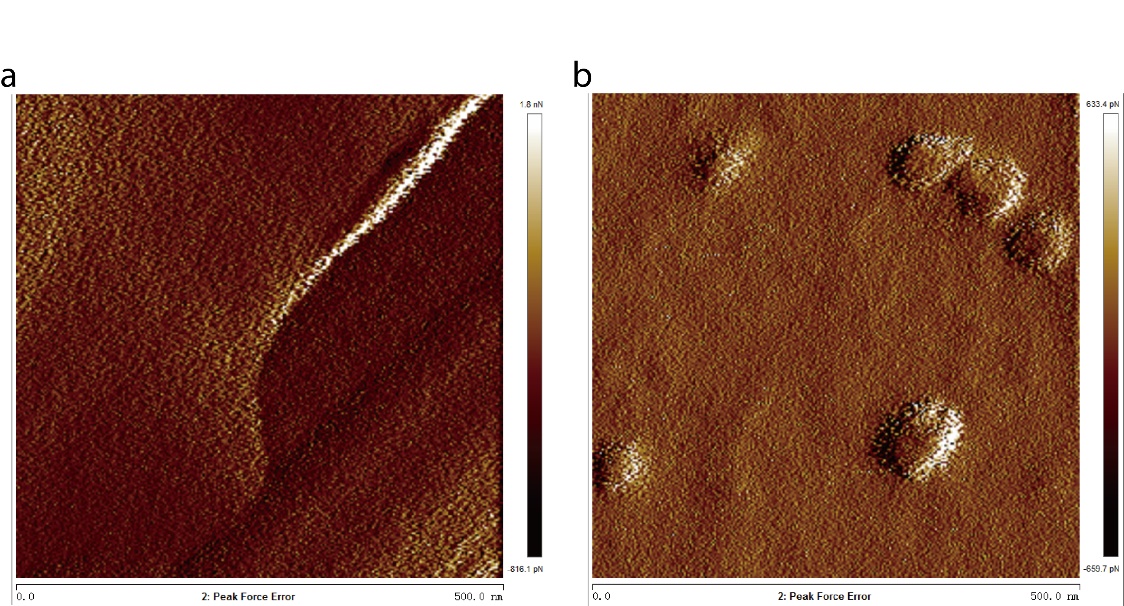


**Fig. S4** Structures of free γ-lactamase and encapsulated γ-lactamase self-assembled in vitro. Representative

images from AFM (data type: peak force error): a) free γ-lactamase, MhIHL-V54L; b) encapsulated γ-lactamase, Enc-I3-01-MhIHL-V54L.


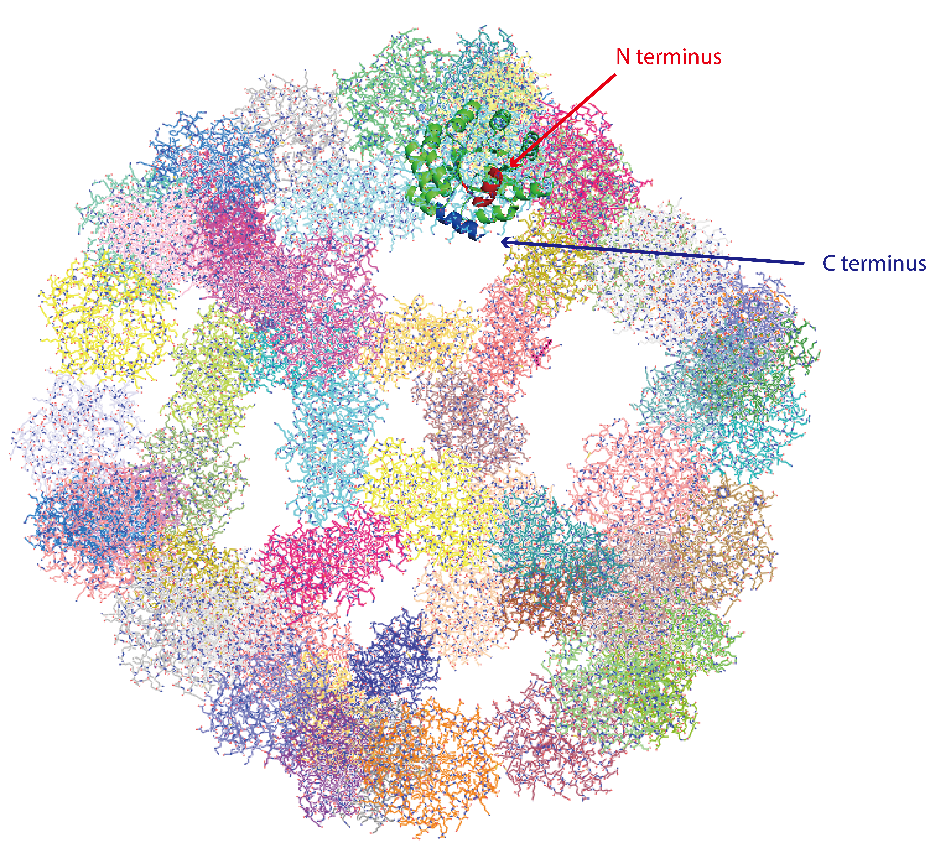


**Fig. S5** Structures of empty protein dodecahedron formed by the engineered ketohydroxyglutarate aldolase from *Thermotoga maritima*. The N terminus of one subunit was marked in red and the C terminus was marked in blue.


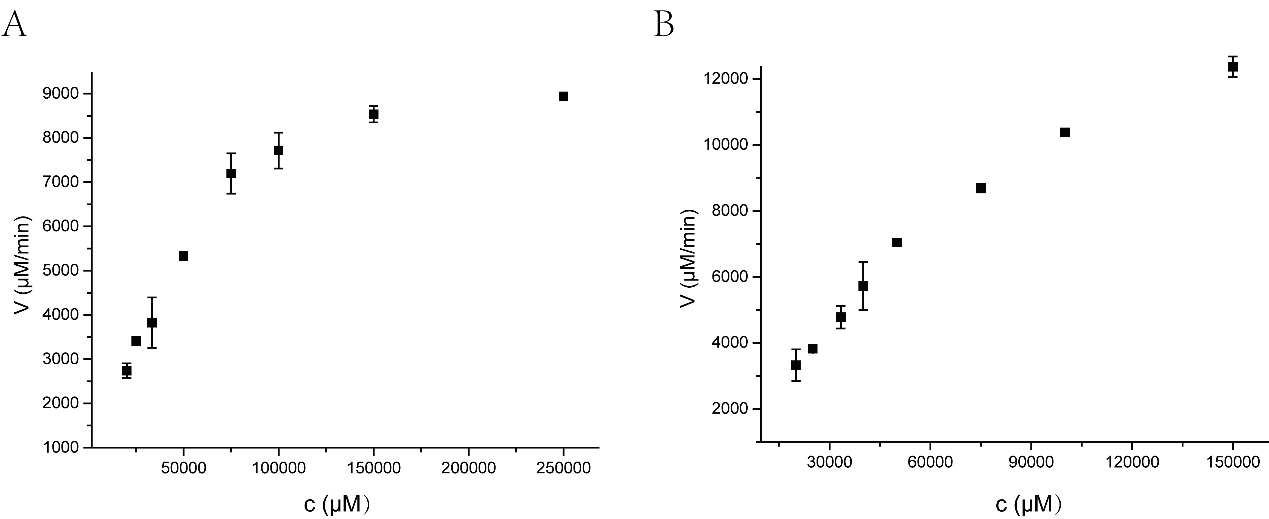


**Fig. S6** Michaelis-Menten plot used to determine the Km and kcat values. Experiments were performed in triplicate (data represent mean +S.D.) A) plot for encapsulated γ-lactamase, Enc-I3-01-MhIHL-V54L. B) plot for free γ-lactamase, MhIHL-V54L;


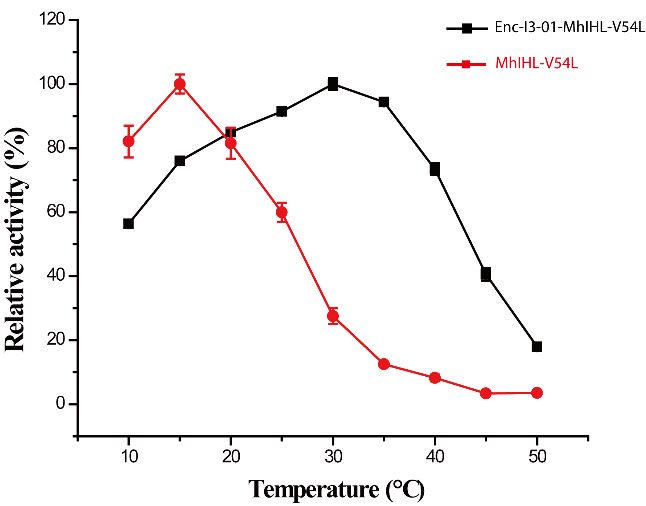


**Fig. S7** Characterizations of the free (red) and encapsulated (+)-γ-lactamases (black). The optimal temperature for the enzymes.


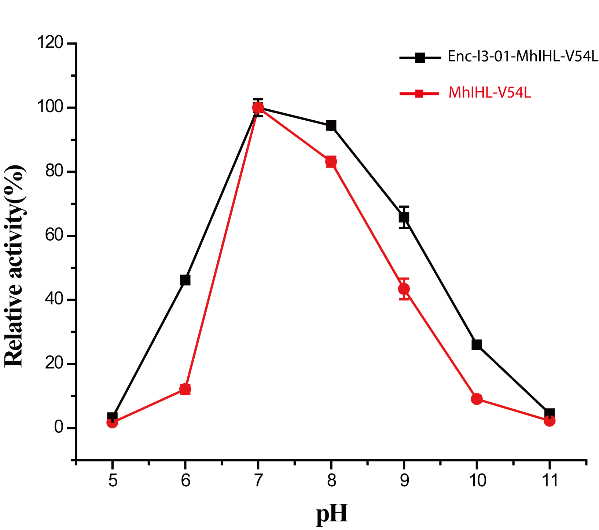


**Fig. S8** Characterizations of the free (red) and encapsulated (+)-γ-lactamases (black). The optimal pH for the enzymes.


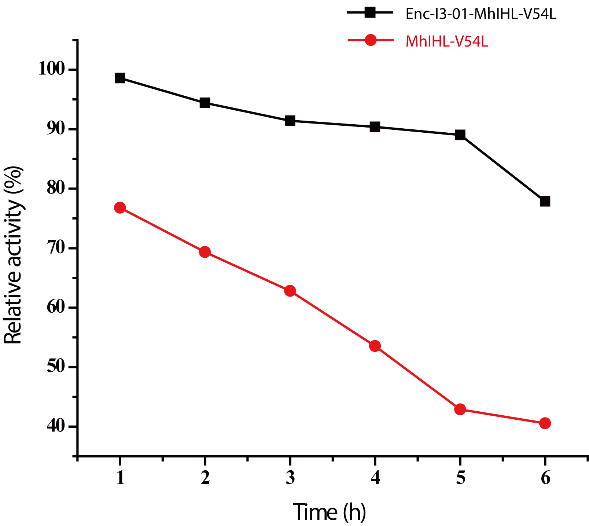


**Fig. S9** Biochemical characterization of free and encapsulated γ-lactamase. Comparison of the stability of the free (red) and encapsulated γ-lactamase (black) in presence of protease (1:10).

**
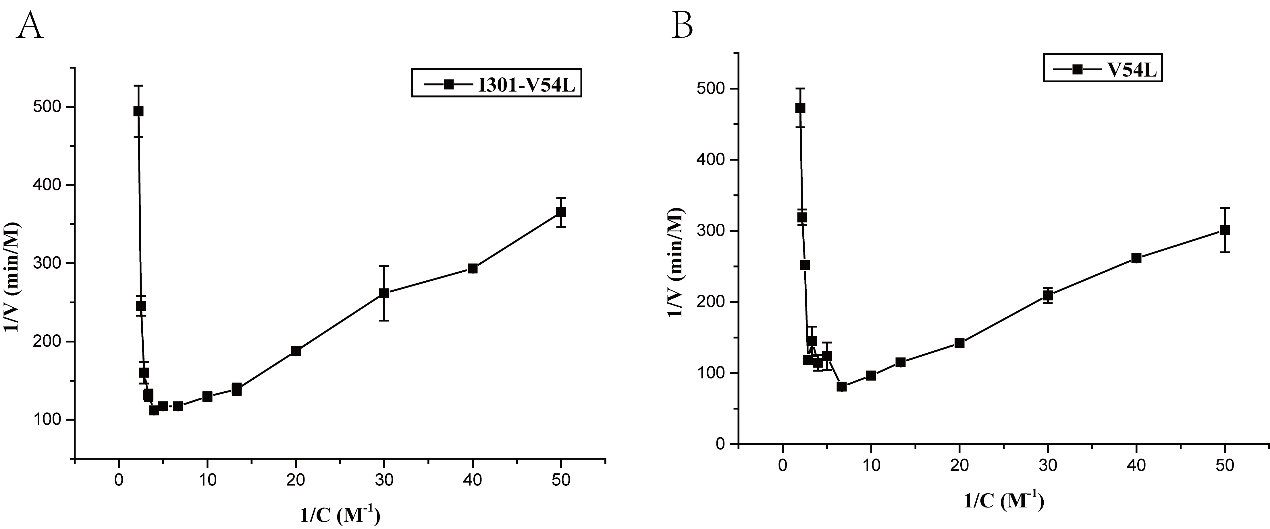
**

**Fig. S10** The Lineweaver–Burk double reciprocal plot for different concentrations of (+)-γ-lactam. A) plot for encapsulated γ-lactamase, Enc-I3-01-MhIHL-V54L. B) plot for free γ-lactamase, MhIHL-V54L;
